# Comparison of Smart Bands with MEMS and Motion Tracking Systems Used in Running †

**DOI:** 10.3390/s25082354

**Published:** 2025-04-08

**Authors:** Andy Stamm, Ronny Hartanto, Noah Becker

**Affiliations:** 1 Faculty of Technology & Bionics, Rhine-Waal University of Applied Sciences, 47533 Cleve, Germany; ronny.hartanto@hsrw.eu; 2 School of Engineering and Built Environment, Griffith University, Nathan 4111, QLD, Australia

**Keywords:** accelerometer, IMU, stride frequency, steps, distance, calories

## Abstract

This research utilizes a self-developed inertial sensor in conjunction with a motion tracking system and four Smart Bands to record a runner’s movements and extract parameters such as step numbers, length, and frequencies at different running speeds. The data obtained were processed to find the number of steps taken. These steps were also recorded by an IMU (inertial measurement unit) and motion tracking system (MoCap—Motion Capturing) and used to compare the different systems and find their agreement at different running speeds. The results show a very strong correlation between the IMU and the motion tracking system with r^2^ = 0.968 at 6 km/h, r^2^ = 0.991 at 8 km/h, r^2^ = 0.991 at 10 km/h, and r^2^ = 0.996 at 12 km/h. The Xiaomi Mi Band 2 showed an r^2^ = 0.861 at 6 km/h, r^2^ = 0.985 at 8 km/h, r^2^ = 0.977 at 10 km/h, and r^2^ = 0.987 at 12 km/h and can therefore be considered a good replacement for an IMU.

## 1. Introduction

Investigating the performance of athletes or amateurs to record training progress is of great interest not only to coaches and athletes but also to amateur runners nowadays. Technology development in recent years has made a large number of devices available for tracking running sessions. Smart bands, for example, are already available at around USD 20 and can record and present results to users. These smart bands could be used to complement or even substitute professional systems such as video camera systems [1], GPS devices [2], and contact force plates [3]. GPSs allow for the very accurate tracking of an athlete but need a direct line of sight to a satellite, which limits them to outdoor use. The manufacturers of smart bands state that their devices are as accurate as professional equipment. While major problems in a running style can be seen and identified by a coach, small problems need the help of technical equipment to identify areas of improvement.

Professional equipment, with its complexity in use and low availability, leads to athletes not being able to monitor each training session at a detailed level. In comparison, smart bands are cheap, easy to use, and therefore available to everyone.

Smart bands are capable of measuring the number of steps taken and use this to calculate the distance traveled and calories burned during a run. The recorded data are usually transferred to a Smartphone where they are locally stored and uploaded to the cloud. Smartphone apps also represent the recorded data graphically and therefore simplify interpretation for the user and allow them to keep a history of old training sessions for comparison. Compared with smart bands, IMUs have been used by many researchers to analyze running and walking [1,2,3,4,5]. This study builds on a previous publication [6], further investigates the data collected from smart bands, and compares these data with the recorded IMU data at different running speeds.

Smart bands are small, light, and easy to use and can be operated without any technical knowledge. These small devices do not hinder or restrict athletes during running and will therefore help to increase the number of monitored training sessions significantly. With sensors and technology, fitted Smart Bands enable the continuous monitoring and recording of physiological and movement-related data. By incorporating components such as accelerometers, gyroscopes, and heart rate sensors, Smart Bands can record an athlete’s physical activity [3,6,7]. Smart bands have been used for interactions with video games [7], vertical impact loading during outdoor running [8], automatic step counting with respect to the level of activity of a person [9], and for differentiation between mental stress and activities [10].

Quantifying athletes’ running information can be conducted using video systems, GPS devices, and motion tracking systems in conjunction with treadmills. Video analysis is used as the gold standard but has some drawbacks, such as (a) parallax errors, (b) inaccuracy in measurements due to unrecognizable reference points, and (c) the extensive time taken to digitize data (frame by frame). Long post-processing is one of the major problems associated with this method and does not allow for post session feedback to be delivered to athletes/coaches. MoCap systems have similar problems, as they are complex to set up and need some post-processing time before the results are made available to athletes/coaches [3].

This manuscript is an extended version of [6], with a significant extension enabled by more data analysis (considering individual speeds for each smart band), new comparisons, new statistics, and more detailed results, allowing us to present more valuable results to the reader.

There are no scientific investigations, to our knowledge, that have validated the accuracy of Smart Bands or Smart Watches against IMUs or MoCap systems. We therefore have the opinion that this research is unique and the first to scientifically investigate the accuracy of Smart Bands in terms of step count and distance run.

This study investigates how well Smart Bands can deliver step count and distance results as performance measures and how accurate these devices are compared to gold standard systems like MoCap, video camera systems, or IMUs. This research uses the recorded tri-axial acceleration of a sacrum-mounted IMU [11], a MoCap system, and compares the findings with the data recorded by four wrist-worn smart bands to find the step count and distance run.

## 2. Materials and Methods

### 2.1. Instrumentation

This study used the SABEL Sense (IMU) [11], which contains a tri-axial accelerometer with a range of ±16 g, a tri-axial gyroscope (2000 degrees per second), and a tri-axial magnetometer. The IMU was set to record at a sampling rate of 100 Hz. A U.N.O. LTX6 Professional treadmill (Beny Sports Germany GmbH, Nuernberg, Germany [12]) was used as a second reference system. The four different Smart Bands used within this study were as follows: Fitbit Alta (Fitbit Inc., San Francisco, CA, USA [13]), Samsung Gear Fit SM-R350 (Samsung Electronics GmbH, Schwalbach/Taunus, Germany [14]), Vidonn X6 (Vidonn Information Technology Co., Ltd., Shenzhen, China [15]), and Xiaomi MiBand 2 (Xiaomi Singapore Pte. Ltd., Singapore, Singapore [16]). These four Smart Bands were chosen as they represent the typical bands sold at this time and their availability in research labs.

The motion tracking system used in this research consists of 8 NaturalPoint OptiTrack Prime 13 cameras (Natural Point Inc., Corvallis, OR, USA [17]) which were calibrated before the experiments were started, and the motion tracking software Motive was also used [18]. The motion tracking system was set to record at a sampling rate of 200 Hz. The IMU was attached at the center of an Optitrack Riged Body Base (Part number MCP1145) with three Optitrack M4 12.7 mm markers (Part number MKR127M4) attached to this base to form a trackable rigid body object.

### 2.2. Data Collection

Six participants (see Table 1) with different experience levels took part in this study, which was approved by the institution’s ethics committee in line with the Helsinki protocol for human research. Data were collected in an indoor lab environment. Each participant used the first low-effort running session as a personal warm up followed by another low-effort, two medium-effort, and one full-effort run. Each run had a duration of 5 min. Low, medium, and full effort were indicated as either 6 km/h–8 km/h–10 km/h or alternatively as 8 km/h–10 km/h–12 km/h depending on the participant’s experience and current training level, and they were selected by the participants.

**Figure 1:**
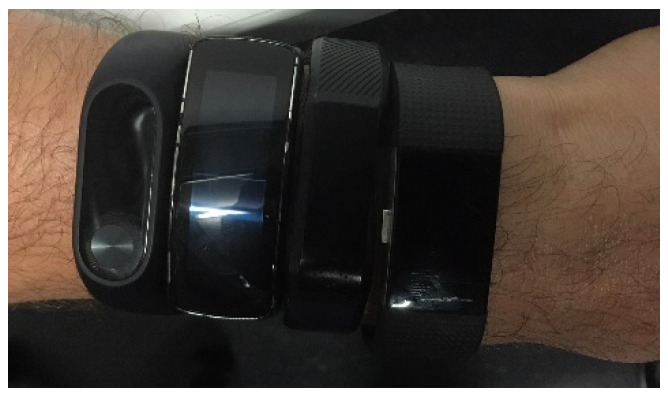
Smart bands and the order in which they were attached to the participants.

In this study, the IMU was attached to the athlete by either using a belt or Velcro when the athlete was using special training pants. The belt/Velcro movement in relation to the skin/bone movement was considered to have only very little influence on the measurements as only acceleration peaks (in running more than 5 g during impact) were used for step detection. These very small movements from the Velcro/belt are in a magnitude so small (less than 0.5 g) in comparison to the foot impact of more than 5 g that these can be neglected as they are not relevant for our peak detection algorithm. The vertical direction, which was aligned with the spine, is represented by a_x_, the mediolateral direction by a_y_, and the anterior–posterior direction by a_z_. Smart Bands were worn on the left lower arm in the following order, starting from the hand: FitBit, Vidonn, Samsung, and Xiaomi (see Figure 1). For data collection, the proprietary software provided by each manufacturer was used. The IMU data were downloaded wirelessly into MATLAB^®^ directly at the end of each running trial, while the treadmill and Smart Band data were recorded manually and transferred into MATLAB^®^. The motion tracking data were exported from MOTIVE as a CSV file and directly imported into MATLAB^®^.

### 2.3. Data Processing

**Figure 2:**
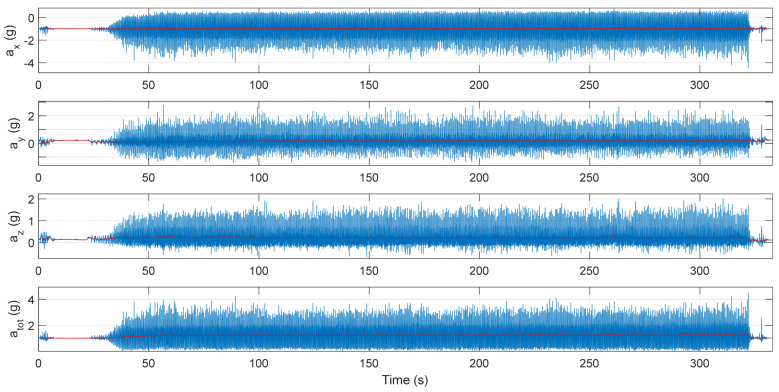
Data recorded by the IMU (blue) with the overlapped gravity component (red).

The IMU-recorded acceleration data were first calibrated before the data analysis was undertaken in MATLAB^®^. The downloaded acceleration data collected by the IMU were first calibrated using a calibration method similar to the method described by Lai et al. [19] before they were high-pass-filtered using a Hamming windowed FIR filter with a 0.5 Hz cut-off frequency as described by Stamm et al. [20]. This was conducted to remove the sensor orientation from the acceleration signal (gravity removal). Figure 2 presents a data set recorded by the IMU (a__tot_—total acceleration) of one running trial (participant 1, blue) with the overlapped filtered gravity component (red).

**Figure 3:**
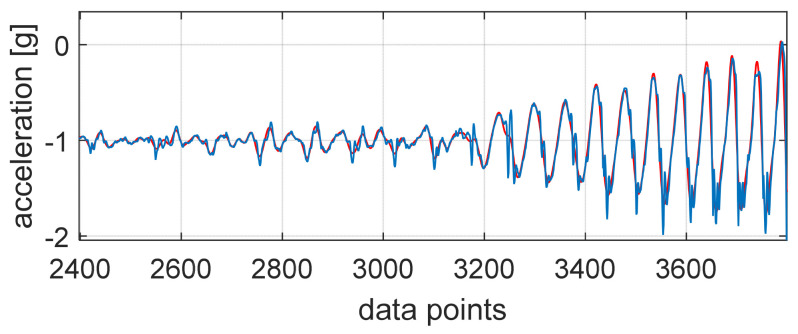
Vertical direction IMU acceleration (blue) with the filtered acceleration data overlaid (red).

The vertical direction was further investigated to find the number of steps taken. This was achieved by applying a peak detection algorithm after the vertical direction data were filtered with a Hamming window FIR filter with a cut-off frequency of 5 Hz. This way, errors in the peak detection process were reduced. Figure 3 presents the vertical direction data (blue) at the start of one low-effort trial with the filtered data overlaid (red), which were used for the peak detection algorithm.

**Figure 4:**
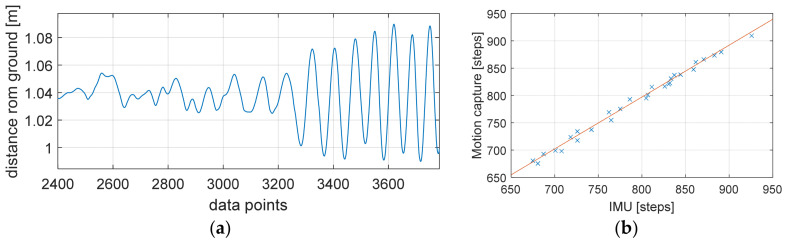
(**a**) Vertical distance recorded by motion tracking system in reference to ground; (**b**) regression analysis of IMU vs. motion tracking system.

**Figure 5:**
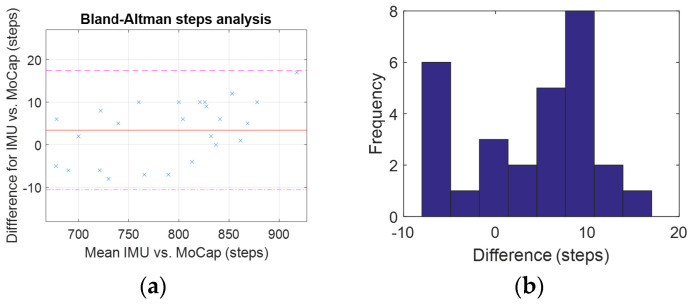
Statistical analysis of IMU vs. motion tracking measurements. (**a**) Bland–Altman analysis. (**b**) Histogram of calculated step differences.

The data recorded by the motion tracking system were imported into MATLAB^®^ for processing. Figure 4a shows the a_y_ channel (which was aligned with the spine) of the imported data from the motion tracking system where the peak detection algorithm could be applied without any further pre-processing. The implemented peak detection algorithm was used to find the number of steps taken for both data sets before they were stored in an array for further analysis (see Table 2). A linear regression analysis (coefficient of determination) comparing the number of steps (all trials) extracted from the IMU and the motion tracking system was conducted to show the alignment of both methods (Figure 4b). Moreover, a Bland–Altman analysis was carried out to compare the IMU and motion tracking measurements (Figure 5). Later, the step data of every smart band were compared with the steps derived from the IMU in a similar way to find the agreement between the two methods.

One key aim of this study was to validate the IMU mounted at the lower back of the participants as being a replacement for a complex MoCap. The results gathered from the comparison of these two systems showed a very strong correlation at all four different measured speeds with r^2^ = 0.968 at 6 km/h; r^2^ = 0.991 at 8 km/h; r^2^ = 0.991 at 10 km/h; and r^2^ = 0.996 at 12 km/h, respectively. It can be seen that over these increases in speeds (from 6 km/h to 12 km/h), the correlation also increased to a value nearly showing perfect correlation. This leads to the conclusion that both systems can detect the number of steps taken with the same accuracy.

Utilizing this knowledge, further analysis was only carried out by comparing the measurements recorded by the different Smart Bands with the IMU-derived data. The results for the Smart Bands were evaluated to be independent from the speed of the participant and trial.

## 3. Results

One trial had to be excluded from the data analysis due to a problem with the IMU. Three further trials showed problems with the step counters of two Smart Bands. This left a total of 29 running trials for further analysis (see those not colored in Table 2) as the problems with the Smart Bands occurred for only one smart band at any time, so data analysis could be carried out for all other Smart Bands. Table 2 presents the steps, and Table 3 presents the distances recorded by all devices for the individual participants and speeds.

Investigating the recorded data further shows that the Vidonn smart band had two outliers at the speed of 8 km/h and that the Samsung band had one outlier at the speed of 12 km/h (marked gray in Table 2). These data points were disregarded for the statistical analysis. It, however, remains unclear why these outliers were recorded by these Smart Bands.

Linear regression analysis in our previous study [6] showed a very strong agreement with a slope of 1.011 and an r^2^ of 0.998 (Figure 4b) between the IMU and the motion tracking system. Bland–Altman analysis was additionally used to find the agreement between these two methods in our previous study. The result showed a bias of 3.86 with an upper limit of agreement of 17.8 and a lower limit of agreement of −9.99 with all data points inside the 95% confidence bounds. The scattering around the bias was even and followed a normal distribution with a skewness of 0.65 and a kurtosis of 2.05. Because of this very strong agreement, further data analysis was only carried out with the number of steps derived from the IMU compared with the individual Smart Bands.

Table 4 presents the results of the different speeds and the different Smart Bands with their individual correlation coefficient and slope.

It can be seen that the correlation coefficient r^2^ changes for each Smart Band at different speeds. For example, the Samsung band has perfect correlation at the slowest investigated speed of 6 km/h, while the correlation decreases with increasing speed, leading to a correlation of only 0.113 at 12 km/h. Compared to this, the Xiaomi band has a correlation of 0.861 at 6 km/h and has an increasing correlation with increasing speeds, leading to a correlation of 0.987 at 12 km/h.

**Figure 6:**
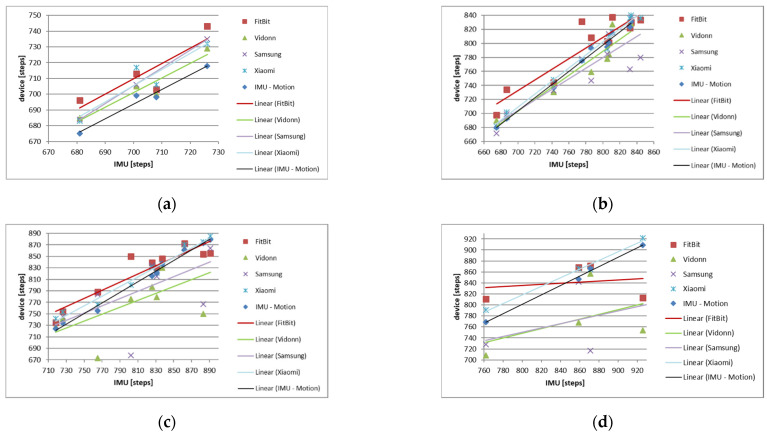
Regression analysis of IMU vs. motion tracking system and Smart Bands at (**a**) 6 km/h, (**b**) 8 km/h, (**c**) 10 km/h, and (**d**) 12 km/h.

Figure 6a presents the trend line for all Smart Bands and the motion tracking system in relation to the IMU at 6 km/h. It can be seen that the slopes of all five investigated devices are similar. Figure 6b presents the slopes at the speed of 8 km/h.

Figure 6c presents the slopes at the speed of 10 km/h with all bands having similar slopes again. Figure 6d presents the slopes at the speed of 12 km/h. This figure confirms that only the Xiaomi band is graphically in line with the IMU-Motion trend line showing a very similar slope. All other bands have different slopes which are not in agreement with the two aforementioned ones.

**Figure 7:**
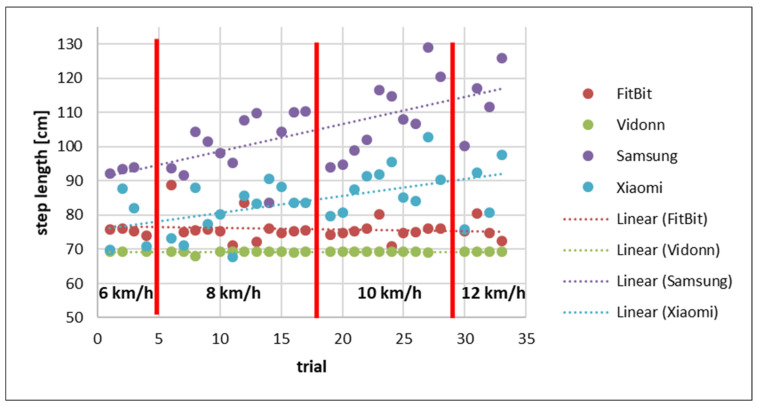
Step length recorded by Smart Bands based on distance measured by Smart Bands sorted by speed.

Figure 7 presents the step length calculated by dividing the distance (recorded from the Smart Bands) by the number of steps taken.

**Figure 8:**
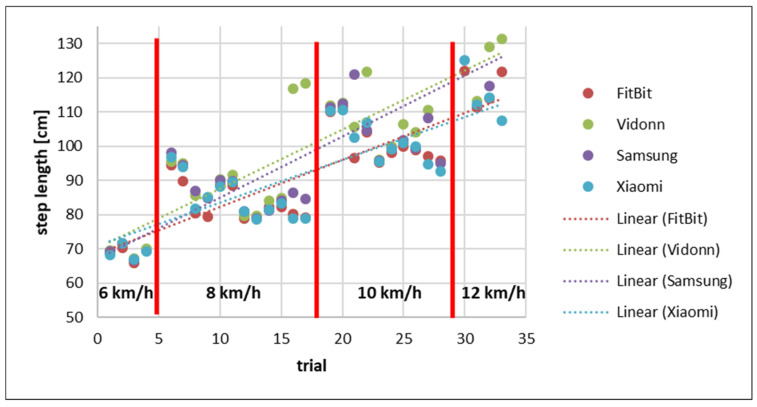
Step length recorded by Smart Bands based on distance measured by treadmill sorted by speed.

Figure 8 presents the step length calculated by dividing the distance (recorded from the treadmill) by the number of steps taken.

## 4. Discussion

This research investigated how well Smart Bands can measure the number of steps taken while running on a treadmill. It utilized a MoCap in conjunction with a self-developed IMU attached to the sacrum of the participant as two reference systems, such as those investigated and compared by [21]. They investigated 231 research articles in conjunction with running and how IMUs are used in running research and found that the majority of research was conducted indoors. Another researcher investigated an IMU attached to the foot of runners and compared the results with a MoCap system [22]. They found Pearson correlations of 0.996 and 0.997 for two different measurements, leading to the assumption that IMUs worn on the foot can be a viable way to assess running kinematics. Additionally, by utilizing an IMU in conjunction with a treadmill, thus obtaining video data as well as data received from track running, [23] investigated how well an IMU could be used to find step counts and energy expenditure. They found an r^2^ of 0.96, which they reported as being in line with similar studies from other researchers. In summary, there have been many studies that compared IMUs with reference systems such as video-based ones or treadmills and found that the accuracy of the IMU-derived results were very strong. This leads to the conclusion that IMU measurements can be used in many sports as a replacement for a gold standard system. The two abovementioned reference systems were also used to conduct a comparison between the estimated step lengths recorded by the Smart Bands in [3,6]. Our study also used IMUs, a treadmill, and a MoCap system to compare our findings with these gold standard systems.

The validation of the four different Smart Bands showed that the Xiaomi MiBand 2 also had a strong correlation of r^2^ = 0.861 at 6 km/h followed by very strong correlations of r^2^ = 0.977 and above for the speeds of 8, 10, and 12 km/h. The FitBit Alta had a good correlation of r^2^ = 0.765 and above for the speeds of 6, 8, and 10 km/h followed by a very weak correlation of r^2^ =0.042 at 12 km/h. The Samsung Gear Fit had a perfect correlation of r^2^ = 1.000 at 6 km/h followed by a strong correlation of r^2^ = 0.748 at 8 km/h, a weak correlation of r^2^ = 0.387 at 10 km/h, and a very weak correlation of r^2^ = 0.113 at 12 km/h. The Vidonn X6 had a very strong correlation of r^2^ = 0.902 at 6 km/h followed by another strong correlation of r^2^ = 0.913 at 8 km/h, a weak correlation of r^2^ = 0.383 at 10 km/h, and a very weak correlation of r^2^ = 0.222 at 12 km/h. A more detailed analysis of the participants’ data was not undertaken as this study was focused on validating the accuracy of Smart Bands rather than the performance of an individual participant.

The comparison of the step lengths recorded by the Smart Bands showed that the FitBit and Vidonn use a constant step length regardless of the speed of the runner. However, the Samsung and Xiaomi Smart Bands show a trend of a dynamically increasing step length with increasing speed. This shows that the recorded distance of the Samsung and Xiaomi bands is more accurate than the recorded distance of the FitBit and Vidonn. Additionally, the comparison with the step length calculated using the distance recorded by the treadmill indicates a larger step length for all Smart Bands with a higher speed.

As the market of Smart Bands is changing quickly, an updated study with newer available Smart Bands should be carried out to investigate how accurately they can count steps. The trend that all investigated Smart Bands show a strong correlation at lower speeds needs to be investigated in more detail in a future study, e.g., by recording at more speeds and smaller steps. It could further be investigated how these Smart Bands perform under normal training conditions and not on a treadmill.

## 5. Conclusions

IMUs have been validated by many researchers (including the authors) to be a good substitute for a MoCap or video system and were therefore used as a standard device to compare the obtained results with [3,6,7,10]. The accuracy of the Xiaomi Smart Band can be considered very strong and therefore allows similar results to be obtained than those obtained by the IMU without the need for operating an IMU or analyzing the obtained data. The Samsung and Fitbit Smart Bands still have a strong correlation with the IMU at medium to lower speeds and therefore are still able to substitute the use of an IMU at these speeds, however, with a lower precision. The Vidonn Smart Band only offers a very strong correlation for the slowest investigated speed and therefore can only replace an IMU at this speed. Since the Samsung and Xiaomi Smart Bands use a greater step length with increasing speeds, the recorded distances are more accurate than the ones recorded by the FitBit and Vidonn.

The growing availability of these small-size and -weight Smart Bands allows for their wider uses for each individual athlete. Nevertheless, there are still large differences in the accuracy of these devices, with some devices being capable of substituting more expensive laboratory equipment such as IMUs or motion tracking systems. A good Smart Band therefore allows athletes or hobby sportspeople to track their training progress relatively accurately for every conducted training session.

This study investigated the accuracy of Smart Bands and showed that they cannot be used as a replacement for sophisticated equipment in its entirety. Only one manufacturer (Xiaomi) showed strong to very strong correlations of r^2^ = 0.861 at 6 km/h and r^2^ = 0.977 at 8, 10, and 12 km/h with the IMU as a reference system. It is therefore suggested to utilize these Smart Bands only for recreational athletes and not as a professional training substitute.

## Figures and Tables

**Table 1:** List of participants, with data on their height, mass, experience, and gender.

Participant Number	Height (cm)	Mass (kg)	Experience	Gender
1	185	82	Novice	Male
2	161	67	Novice	Male
3	167	57	Novice	Female
4	176	89	Recreational	Male
5	171	62	Recreational	Male
6	166	65	Triathlete	Female

**Table 2:** The recorded steps of all different devices, speeds, and participants. X indicates a problem with the measurement; gray-colored measurements are identified as outliers and therefore not considered in the analysis.

Runner	Trial	Speed km/h	Steps Recorded					
			IMU	Motion Tracking	FitBit	Samsung	Vidonn	Xiaomi
1	1	8	675	680	698	672	690	682
	2	8	687	693	734	699	695	702
	3	10	726	734	754	745	742	753
	4	10	718	724	735	729	728	742
	5	12	762	769	811	728	708	791
2	1	8	786	793	808	747	759	795
	2	8	775	775	831	778	X	776
	3	10	802	X	850	678	776	800
	4	6	701	699	713	706	705	717
	5	6	681	675	696	684	685	683
3	1	6	726	718	743	735	729	732
	2	6	708	698	703	X	700	706
	3	8	742	737	744	733	731	748
	4	8	X	X	745	735	720	737
	5	10	765	755	788	784	673	766
4	1	8	811	815	837	816	827	816
	2	8	833	831	830	837	828	840
	3	10	862	861	872	866	864	870
	4	10	837	837	846	836	830	837
	5	12	871	866	871	717	857	865
5	1	8	807	801	801	813	785	810
	2	8	805	795	803	785	778	792
	3	10	831	821	830	815	779	821
	4	10	826	816	839	834	797	831
	5	12	859	847	868	842	768	866
6	1	8	832	823	822	763	565	836
	2	8	844	838	833	780	557	836
	3	10	883	873	854	767	750	875
	4	10	891	879	855	864	X	885
	5	12	926	909	813	611	754	922

**Table 3:** The recorded distances of all different devices, speeds, and participants. X indicates a problem with the measurement; gray-colored measurements are identified as outliers and therefore not considered in the analysis.

Runner	Trial	Speed km/h	Distance Recorded [m]				
			Treadmill	FitBit	Samsung	Vidonn	Xiaomi
1	1	8	660	620	630	478	500
	2	8	660	550	640	482	500
	3	10	830	560	700	514	600
	4	10	820	550	690	505	600
	5	12	990	610	730	490	600
2	1	8	650	610	780	517	700
	2	8	660	630	790	X	600
	3	10	820	640	670	538	700
	4	6	490	540	650	488	500
	5	6	490	530	640	475	600
3	1	6	490	560	690	X	600
	2	6	490	520	X	485	500
	3	8	660	560	720	507	600
	4	8	660	530	700	499	500
	5	10	820	600	800	467	700
4	1	8	660	700	880	573	700
	2	8	660	600	920	574	700
	3	10	830	700	1010	599	800
	4	10	830	600	960	575	800
	5	12	970	700	840	594	800
5	1	8	660	610	680	544	733
	2	8	660	600	820	539	700
	3	10	830	620	880	540	700
	4	10	830	630	890	553	700
	5	12	990	650	940	532	700
6	1	8	660	620	840	391	700
	2	8	660	630	860	386	700
	3	10	830	650	990	518	900
	4	10	820	650	1040	X	800
	5	12	990	590	770	522	900

**Table 4:** The results of the linear regression analysis (steps) derived from the IMU vs. the motion tracking system and the individual Smart Bands clustered at different running speeds.

Device	IMU—6 km/h		IMU—8 km/h		IMU—10 km/h		IMU—12 km/h	
	r^2^	Slope	r^2^	Slope	r^2^	Slope	r^2^	Slope
Motion tracking	0.968	1.040	0.991	1.058	0.991	1.078	0.996	1.163
FitBit	0.765	0.786	0.845	1.116	0.854	1.205	0.042	0.419
Samsung	1.000	0.881	0.748	0.990	0.387	0.611	0.113	0.291
Vidonn	0.902	0.967	0.913	1.072	0.383	0.634	0.222	0.516
Xiaomi	0.861	0.836	0.985	1.062	0.977	1.167	0.987	0.996

## Data Availability

The data used for this study can be requested from the corresponding author.
